# The impact of emotional contagion on workplace safety: Investigating the roles of sleep, health, and production pressure

**DOI:** 10.1007/s12144-021-01616-8

**Published:** 2021-03-18

**Authors:** Laura Petitta, Tahira M. Probst, Valerio Ghezzi, Claudio Barbaranelli

**Affiliations:** 1 Department of Psychology, Sapienza University of Rome, Via dei Marsi, 78, 00185 Rome, Italy; 2 Washington State University Vancouver, 14204 NE Salmon Creek Avenue, Vancouver, WA 98686-9600 USA

**Keywords:** Emotional contagion, Production pressure, Sleep disturbances, Health problems, Workplace accidents and injuries

## Abstract

Using emotional contagion theory and the Job Demands-Resources model as a theoretical foundation, we tested the proposition that higher levels of contagion of anger (i.e., a demand) vs. higher levels of contagion of joy (i.e., a resource) will be associated respectively with more vs. fewer sleep disturbances and health problems, which in turn are related to more workplace accidents and injuries. Moreover, we examined the moderating impact of production pressure (i.e., a contextual demand) on the relationship between emotional contagion and employee poor sleep and health. Data from 1000 employees in Italy showed that the conditional indirect effects of contagion of anger, but not of joy, on accidents and injuries via sleep and health problems were intensified as levels of production pressure increased. Furthermore, contagion of anger was positively associated with both sleep disturbances and health problems whereas contagion of joy was negatively related to only sleep disturbances. These findings suggest that the effect of anger that employees absorb during social interactions at work likely persists when coming at home and represents an emotional demand that impairs the physiological functions that regulate restorative sleep and energies recharging; and, this effect is even stronger among employees who perceived higher levels of organizational production pressure.

Sleep disturbances are typically thought of as something that primarily only concerns the affected individual; however, existing evidence suggest that sleep disturbances also have serious consequences for organizations and the economy (Hafner et al., 2016). For example, lack of adequate sleep adversely impacts cognitive performance, injuries and workplace productivity, with a lack of sleep leading to more traffic accidents, industrial accidents, medical errors and loss of work productivity (Nuckols et al., 2009; Pack et al., 1995).

According to the Centers for Disease Control and Prevention (CDC), more than one third of American adults do not get enough sleep on a regular basis (Liu et al., 2016), and insufficient sleep also concerns other industrialized countries such as the United Kingdom, Japan, Germany, and Canada (National Sleep Foundation, 2013). Estimates (e.g., Hafner et al., 2016) of the annual economic losses due to insufficient sleep on labor productivity across different countries range from $50 billion in the United Kingdom to a staggering $411 billion in the United States. Such costs are alarming given evidence of the rising rates of insufficient sleep worldwide (Hafner et al., 2016). For example, within Italy where the current study took place, a recent prevalence study using a nationally representative sample found that 30% of respondents reported experiencing insomnia (defined as at least one sleep problem several times per week for more than a year with daytime consequences; Leger et al., 2008). While individuals spend approximately one-third of their time at work, and one-third asleep, recent meta-analytic findings (Litwiller et al., 2017) still report the absence of a sufficient number of studies related to employee sleep and safety outcomes that consequently prevented conclusive answers about how safety constructs relate to sleep. Furthermore, while the literature provides cumulative and meta-analytic evidence of the *consequences* of sleep disturbances on safety outcomes (e.g., Kuehli et al., 2014), the empirical inquiry on individual and situational determinants of sleep disturbances and health problems that adversely impact safety is still scarce.

Our study sought to fill this gap by examining individual and contextual antecedents (specifically, emotional contagion at work and organizational production pressure) and safety-related consequences (specifically, workplace accidents and injuries) of sleep disturbances and health problems. An accident can be defined as an unplanned and uncontrolled event that led to: injury to persons, damage to property, or some other loss to the company (European Agency for Safety and Health at Work; EU-OSHA, 2019). Conversely, an injury refers to the physical damage occurred as a consequence of an accident (Barling & Frone, 2004). Using the emotional contagion literature (Hatfield et al., 1993) and the Job Demands-Resources (JD-R) model (Demerouti et al., 2004) of stress as a theoretical foundation, we test the proposition that higher levels of contagion of anger (i.e., a job demand) vs. higher levels of contagion of joy (i.e., a job resource) will be associated respectively with more vs. fewer sleep disturbances and health problems, which in turn are related to higher rates of workplace accidents and injuries. Moreover, we test the moderating impact of organizational production pressure (i.e., a contextual demand) on the relationship between emotional contagion of positive and negative emotions, and subsequent employee sleep disturbances and health problems. That is, individuals under high pressure to produce may be even more sensitive to the effects of anger absorbed during social interactions at work, thus resenting the consequences of negative emotions on their sleep and health more so than individuals under low pressure to produce (i.e., an expected exacerbating effect). Similarly, high production pressure may impede the capacity of joy absorbed by others to help employees recharge their energies and enact health-enhancing behaviors such as restorative sleep more so than employees exposed to low production pressure (i.e., attenuating effect).

Taken together, this study has two main aims, each contributing to the extant literature in a unique way. The first aim was to examine whether and to what extent emotional contagion of positive/negative emotions at the workplace may play a role in increasing or decreasing experienced problems in sleep and health as well as safety outcomes. Unfortunately, while the effects of individual factors, such as personal and socio-demographic factors (e.g., financial concerns, provision of unpaid care, presence of children, gender, age, and marital status), on sleep disturbances and related workplace accidents are well documented (Hafner et al., 2016), no research to date has examined whether the contagion of emotions that employees experience at the workplace has a similar relationship. As a result, little is known about the relationship between sleep problems and other relevant and pervasive individual variables (either than socio-economic or demographic factors) that employees experience at work and that they bring home, and which affect their overall health functioning to the point of interfering with their ability to complete the job task safely (Barnes, 2012; Martin, 1983). Indeed, Burgard and Ailshire (2009) found that being frequently bothered or upset at work was predictive of poorer sleep quality. The current study goes beyond the study of affect-related factors that occur solely intrapsychically (e.g., anxiety) and focuses on contagion of discrete basic emotions as an emotion-related factor with a strong social component which can influence both health (e.g., Le Blanc et al., 2001) and relevant safety outcomes (i.e., workplace injuries, accidents). Understanding emotional contagion as an antecedent of sleep and health and subsequent safety outcomes is important because it would allow us to develop effective emotion management interventions including knowledge on how social interactions contribute to shape emotional life of employees and their subsequent health and sleep status, and the likelihood of experiencing accidents/injuries at work.

The second aim was to provide an empirical examination of the interplay between individual (i.e., emotional contagion of joy/anger) and contextual (i.e., production pressure) influences on employee sleep disturbances and health problems. Specifically, we examine how the contagion of positive/negative emotions at work affects employee sleep and health within the context of the employee’s perception of an organizational pressure to produce. Figure 1 presents an overview of our overarching conceptual model. As can be seen, we expect a moderated mediation relationship between contagion of positive/negative emotions, production pressure, sleep and health problems, and poor safety outcomes (i.e., accidents, injuries). In doing so, the current study considers simultaneously the role of individual-related job demands (i.e., emotional contagion of anger) and context-related job demands (i.e., production pressure) as predictors^1^ of safety accidents and injuries. In so doing, we extend the JD-R model of health impairment processes as mechanisms through which job demands and resources relate to safety outcomes (Nahrgang et al., 2011) by examining emotion-related job demands (i.e., emotional contagion of anger) and job resources (i.e., emotional contagion of joy) that employees cannot easily “turn off” once they go home, and that likely affect their sleep and health (Demerouti et al., 2004). Furthermore, extending the JD-R model of safety through health impairment (Nahrgang et al., 2011) to include the role of emotions, as well as their interaction with context-related production pressure, increases our ability to account for unsafe performing of job tasks and related accidents/injuries under time pressure.

**Fig. 1:**
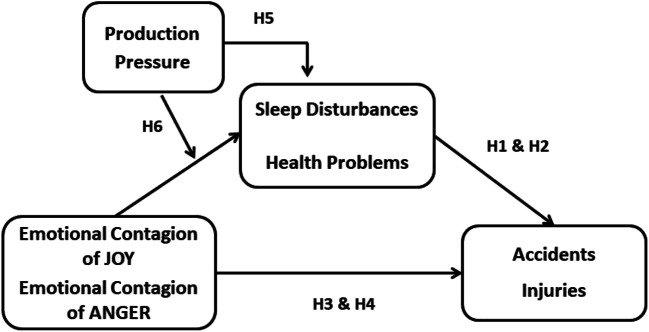
Conceptual mediation moderation model

Below we present our review of the literature by introducing first sleep quality and health condition as independent variables and as proximal predictors of safety outcomes, whereas emotional contagion is addressed as an additional independent variable and a more distal predictor of safety outcomes. We begin by considering sleep deprivation and health problems as antecedents of workplace accidents and injuries. Next, we introduce emotional contagion of positive (i.e., joy) and negative (i.e., anger) emotions as individual antecedents of sleep and health problems, and review literature on the relationships between emotional contagion, sleep and health, and poor safety outcomes (i.e., accidents and injuries). Finally, we introduce production pressure as an organizational antecedent of sleep and health problems and argument its moderating role of the relationship between emotions and sleep and health.

## Sleep Disturbances, Health Problems and Poor Safety Outcomes

Sleep disturbances usually refer to difficulties with initiating or maintaining sleep (Magnusson Hanson et al., 2014). Literature (Hafner et al., 2016, p.2) suggests that “[l]ack of adequate sleep can affect judgment and the ability to process and retain information adequately, and can increase the risk of serious or fatal accidents and injury.” While there are some individual differences in what constitutes “adequate sleep”, researchers generally agree that adults should obtain between 7 and 9 h of sleep each night for optimal health outcomes (Hafner et al., 2016). Specifically, individuals obtaining these levels of sleep nightly experience the lowest risks for all-cause mortality, whereas those who sleep for shorter or longer periods have significantly higher mortality risks (e.g., Vgontzas et al., 2010). Interestingly, negative outcomes are associated with both insufficient sleep and sleeping *more* than the recommended seven to nine hours. While it seems that both extreme ends of sleep distribution patterns really matter for predicting adverse outcomes, the literature also suggests that the link between short sleep and negative outcomes is more direct whereas the effects of long sleep on mortality risks might be driven by underlying chronic health problems (Hafner et al., 2016, p.2).

In the current study we focus on the effects of both sleep disturbances and health problems on poor safety outcomes. Specifically, health problems involve psychosomatic symptoms such as headache and back problems that employees report to have experienced (Hanisch, 1992) and which in our study *do not* include sleep problems. Given that insufficient sleep may cause (i.e., is an antecedent) negative health outcomes (e.g., Hafner et al., 2016) as well as be a symptom (i.e., is a consequence) of underlying health problems (e.g., National Sleep Foundation, 2018), we consider sleep disturbances and health problems as correlates and differential indicators of an overarching psycho-physiological condition of an individual.

As noted above, poor safety outcomes depend upon cognitive- and affect-related factors. For example, research demonstrated that cognitive failures (i.e., cognitively based mistakes or errors in the performance of an action that an individual is normally capable of completing; Martin, 1983) occur not as a result of an inability to complete a task, but rather are due to other variables which interfere with the ability to complete the task. According to Barnes (2012), poor sleep quality and quantity can adversely affect the self-regulatory capacity of employees. In particular, diminished self-regulation can decrease cognitive performance (e.g., alertness, directing and controlling attention, and risk analysis). According to the National Sleep Foundation (2018), sleepiness and fatigue of workers may impair their reaction time, judgment and vision, create problems with information processing and short term memory, and decrease performance, vigilance and motivation. Furthermore, 37% of American adults say they are so tired during the day it interferes with daily activities. Consistent with that, meta-analytic findings (Kuehli et al., 2014) demonstrated that sleep problems are a potential risk factor for work injuries and that workers with sleep problems have a 1.62 times higher risk of being injured than workers without sleep problems. Of particular note, about 13% of work injuries can be attributed to sleep problems.

Consistent with the above arguments, we expect to find the following:

- *Hypothesis 1:* Sleep disturbances will positively predict workplace accidents (1a) and injuries (1b).
- *Hypothesis 2:* Health problems will positively predict workplace accidents (2a) and injuries (2b).

## Emotional Contagion at Work

Emotional contagion (EC; Hatfield et al., 1993) is an unintentional process by which individuals detect and absorb the emotions of others with whom they interact (Hatfield & Rapson, 1998). Research has found that people tend to automatically mimic the facial, vocal, postural, and behavioral emotional cues of others, resulting in them “catching” the emotions of those individuals (Hatfield et al., 1993; p.3). Thus, emotional contagion refers to an exchange of emotions and feelings occurring during interpersonal social interactions (Hatfield et al., 1993). This joint absorption and reflection of the emotional cues portrayed by others occurs subconsciously facilitated by the Mirror Neuron System mimicking the brain activation pattern underlying an emotional stimulus (Iacoboni, 2009; Rizzolatti & Sinigaglia, 2008). Although the activation of emotional contagion occurs involuntarily and automatically, individuals become consciously aware of this emotional exchange when the neocortex receives the emotional signal milliseconds thereafter (LeDoux, 2002). As such, like an epidemic, emotional contagion can spreads throughout large social gatherings (Hatfield et al., 2012) and among employees interacting at work (Petitta & Naughton, 2015).

Although the process of emotional contagion is inherently social in nature, the present paper takes an individual-level perspective of emotional contagion, operationalized as the employee’s experience of feeling an emotion that has been absorbed from interacting with others at work. Furthermore, as recommended by Doherty (1997), we focus on the absorption of specific basic emotions (i.e., joy and anger), rather than simply assessing the general tendencies of an employee to absorb the affective cues (i.e., emotions, feelings, moods) of others (Hatfield & Rapson, 1998). We purposefully focus on the contagion of joy and anger, because these basic and discrete emotions have been shown to be universally experienced and displayed by all humans (Ekman, 1999), thus increasing the likelihood of generalizability of the research findings and applications. Finally, we specifically examine the impact of emotional contagion experienced in the workplace, rather than more generally experienced in everyday situation (Doherty, 1997; Hatfield & Rapson, 1998). In this way, we can specifically test the effect of such workplace emotional contagion on sleep and health outcomes without these effects being co-mingled with other affect-laden experiences (e.g., anger absorbed by spouse at home).

## Sleep, Health and Safety Outcomes of Emotional Contagion

Meta-analytic research by Nahrgang et al. (2011) found that emotion-related job demands (e.g., emotionally demanding relationships) impair employees health, which in turn prevents them from working safely. Furthermore, job resources exerted a complementary effect such as curbing job demands and fostering employee wellbeing, thus providing them the energies to work safely. Job demands refer to psychological (e.g., *emotional* stressors), social, or organizational aspects of the job that require sustained physical and/or psychological effort and are therefore associated with certain physiological and/or psychological costs (e.g., poor sleep and health) for the employee (Bakker & Demerouti, 2007). An example of emotion-related job demands may be an emotionally demanding interaction with customers or clients (Bakker & Demerouti, 2017) from whom employees may absorb anger of frustration, which they retain when coming back at home. Similarly, job resources refer to psychological (e.g., *emotional* assets), social, or organizational aspects of the job that are functional in achieving work goals, stimulate personal growth and development, thus enhancing physical and/or physiological energies (e.g., quality sleep and well-being) of the employee (Bakker & Demerouti, 2007). As such, both job demands and resources can be emotion-related factors that originate during social interaction at work. Therefore, we propose that emotional contagion may be one of the *social*-related aspects that exerts its effects on employee physiological and/or psychological condition (e.g., sleep disturbances and health problems). Specifically, positive emotions (e.g., joy) allow people to operate at an optimal condition, stay positive and pleasant, and maintain great energy (Andrieş, 2011). Thus, contagion of positive emotions (e.g., joy) may serve as a job resource by synchronizing opportunities, social bonding, and cooperation (Hess & Fischer, 2014). Conversely, negative emotions (e.g., anger) are associated with unpleasant, dysfunctional operating conditions (Andrieş, 2011). Thus, negative emotions are demanding and potentially associated with poor wellbeing. As such, contagion of negative emotions (e.g., anger) may have detrimental consequences and thus qualifies as a job demand by depleting psychological resources (Barsade, 2002).

As noted earlier, the psychosocial environment at work influences sleep duration and strained relationships in the workplace may trigger sleep loss (Hafner et al., 2016; Health & Safety Executive, 2018). In one of the first studies to examine the link between stressful experiences (both at home and at work) and sleep outcomes, Burgard and Ailshire (2009) found that being frequently bothered or upset at work was predictive of poorer sleep quality. While no study has examined the effects of emotional contagion of anger on employee sleep and health, we can speculate that the effect of anger that employees absorb during social interactions at work likely persists when coming at home. In turn, this persistence represents an emotional demand/stressor that impairs the body to switch from the activating sympathetic system to the calmer parasympathetic nervous system that helps restoration (e.g., sleep) and overall health-related energy recharging (Christensen et al., 2004). On a different yet related note, empirical evidence (Ong et al., 2013) suggests that the experience of joy and happiness and relatively stable high levels of positive emotions may be conducive to improved sleep.

Previous conceptual contributions from Krauss (Krauss et al., 2003) and Mullins (Mullins et al., 2014) propose a framework of relations among job demands, sleepiness and its consequences for safety, suggesting that job demands such as work load and time pressure can increase sleepiness in the workplace which, in turn, impairs an appropriate and safe processing of the work task and causes unwanted behavioral manifestations (e.g., accidents, injuries). We build on this approach and further expand this conceptual framework by including emotion-related job demands. Specifically, using the JD-R model of stress and emotional contagion literature as a theoretical foundation, we test the proposition that higher levels of contagion of anger (i.e., a demand) will be associated with greater levels of subsequent sleep and health problems, while higher levels of contagion of joy (i.e., a resource) will be associated with lower levels of sleep and health problems. According to the Broaden-and-build theory (Fredrickson, 2001), positive emotions (e.g., joy) not only broaden attention and action but also build physical and social resources. Conversely, negative emotions (e.g., anger) interfere with cognition and narrow the scope of attention and the pursuing of the broader array of thoughts and actions.

Safety literature (Gilboa et al., 2008) suggests that negative emotions (i.e., anger, frustration, anxiety) narrow employees’ attention and subsequent carrying out of work in a safe manner, thus increasing the number of accidents they experience. Similarly, negative emotions may narrow perceptual focus thus causing individuals to miss important performance-related cues and act without considering the consequences of their actions (Shoss & Probst, 2012). In the current study, we expect a similar effect such that the contagion of anger (i.e., a demand) will interfere with employees energies and prevent them from behaving safely and leading them to experience more workplace accidents and injuries.

Accordingly, we argue that contagion of joy (i.e., a resource) will energize employees and help them to avoid safety hazards and experience less accidents and injuries. Indeed, a study from Petitta and colleagues (Petitta et al., 2019) found that emotional contagion of joy and anger among employees exert indirect effects (respectively, negative and positive) on the likelihood of experiencing workplace accidents through cognitive failures.

Taken together, our arguments provide an overarching framework for including both positive and negative emotions and their contagion in the JD-R model as conjoint predictors of sleep and health problems, and related poor safety outcomes. Therefore, we hypothesize that:

- *Hypothesis 3:* Emotional contagion of joy absorbed from others negatively predicts (3a) workplace accidents and (3b) injuries, both directly and indirectly via sleep disturbances and health problems.
- *Hypothesis 4:* Emotional contagion of anger absorbed from others positively predicts (4a) workplace accidents and (4b) injuries, both directly and indirectly via sleep disturbances and health problems.

## The Role of Production Pressure as a Contextual Moderator

Production pressure can be defined as a set of job demands related to the attainment of “operational goals for the purpose of increasing organizational profits and/or efficiency” (Probst & Graso, 2013, p. 581). The changing nature of today’s work in advanced industrial countries (e.g., increased pace of technological change) implies the intensification of work and increased work pressure (Gallie, 2005). A growing body of literature suggests that high levels of work pressure can lead to excessive efforts to achieve production goals (e.g., Meijman & Mulder, 1998) at the expense of safety. That is, production pressure (e.g., being pressed to work faster/harder) increases employees tendency to circumvent safety rules (e.g., Rundmo et al., 1998; Zohar & Luria, 2005) by enacting risky patterns of behavior in order to meet productivity requirements (Han et al., 2014). Specifically, organizations setting high production standards may prioritize performance and undervalue safety aspects (Zohar & Luria, 2005), thus causing employees to legitimize undertaking shortcuts and deviating from safety rules in order to meet productivity goals (e.g., Kaminski, 2001; Keren et al., 2009).

Notably, previous studies demonstrated that production pressure not only negatively affects safety outcomes but some evidence suggests that work pressure to achieve profit goals represent a type of job demand that is highly detrimental to mental and self-reported physical health (Gallie, 2005; Silla & Gamero, 2013). While a growing body of literature demonstrates that production pressure negatively affects safety outcomes, there is only initial evidence suggesting that work pressure to achieve profit goals represent a type of job demand that is likely to be highly detrimental to mental and self-reported physical health (Gallie, 2005; Silla & Gamero, 2013). Moreover, even less is known regarding the potential impact of organizational demands to work faster and more intensely (i.e., production pressure) on employees sleep disturbances.

Consistent with the JD-R approach to the study of employee wellbeing and safety (Demerouti et al., 2004; Kühnel et al., 2012; Nahrgang et al., 2011), the current study proposes production pressure as a job demand related to organizational aspects of the job that requires sustained physical and/or psychological effort and is therefore associated with certain physiological and/or psychological costs for the employee (Bakker & Demerouti, 2007), such as health problems and sleep disturbances.

Although there is as yet no empirical research specifically examining the relationship between production pressure and sleep disturbances, we argue that perceived production pressure places an ongoing demand to think about achievement strategies that employees cannot easily “turn off” once they go home (Demerouti et al., 2004), thus having their minds race with thoughts instead of shutting down at night and obtaining restorative sleep. Based on earlier research suggesting that increased production pressure might impair employee health, it can be argued that sleep disturbances, too, can be swayed by work intensity and time pressure.

On the basis of these arguments, we hypothesize that:

- *Hypothesis 5:* Higher levels of production pressure will predict higher subsequent sleep disturbances (5a) and health problems (5b).

Although the review of the empirical literature above indicates that production pressure is related to reduced wellbeing, until recently, there has been no attempt to evaluate the extent to which such pressure is related to the contagion of emotions absorbed by other at work, or to explain the interplay between emotional contagion and organizational pressure to produce in explaining employees’ health and sleep activities. Consistent with our theoretical framework rooted into the JD-R model of stress and safety outcomes (Nahrgang et al., 2011), we consider production pressure as an organizational demand for the explanation of employee sleep and health problems, and subsequent work accidents and injuries.

According to the JD-R model, job demands may interact with each other (Bakker & Demerouti, 2017) and exert a cumulative effect on employee wellbeing. Therefore, as with emotional contagion of joy and anger, we not only expect a direct effect of production pressure on sleep and health problems, but also a moderation effect. That is, a higher production pressure is likely to exacerbate the effect of anger absorbed by employees at work (i.e., emotional contagion of anger) on the employee sleep and health problems. Specifically, it can be speculated that employees’ sympathetic nervous system not shut down because the contagion of anger makes them overly worried and upset (Christensen et al., 2004). Additionally, organizational pressure to work quickly and to systematically achieve stringent deadlines sets an ongoing demand to employees’ brain to remain hyperactive, thus amplifying the effects of negative emotions on the action-related sympathetic nervous system and impeding the switch to the calmer parasympathetic nervous system associated with rest (e.g., sleep). Conversely, considering the moderating effects of production pressure on the link between emotional contagion of joy and sleep and health problems, we expect an attenuating effect of production pressure such that the employees’ mind remains fast-paced and activated by organizational time pressure, which impedes the experience of joy absorbed by others to activate the calm-related parasympathetic nervous system and to fortify health-enhancing behaviors such as restorative sleep (Ong et al., 2013).

Consistent with the above arguments, we have reason to expect the following:

- *Hypothesis 6*: High levels of production pressure will magnify the positive relationship between emotional contagion of anger and sleep disturbances (6a) and health problems (6b). High levels of production pressure will attenuate the negative relationship between emotional contagion of joy and sleep disturbances (6c) and health problems (6d).

## Method

### Participants and Procedure

The sample consisted of *N* = 1000 employees in Italy. Fifty percent of respondents were male, 49.2% female, with .8% leaving the gender item blank. The mean age of participants was 43.28 years (*SD* = 13.5). The average employee tenure with the current position was 12.3 years (*SD* = 11.02). Sixteen percent were blue collars, 52.3% were white collars, 11.1% were supervisors, while 22.7% held a managerial role. Organizations were recruited from the following industry sectors: health care (13.2*%*); education (8.5%); manufacturing (3.8*%*); transportation (3.6%); communication & technology (15,6%); military (3.4*%*), construction (2.7*%*), agriculture (.9%), services (28.4*%*), and 19 % did not specify the sector. Thirty-two percent of respondents worked for organizations in the public sector while 68.1% worked for private companies.

The research team approached administrators within each organization to request their organization’s participation in the study. Paper and pencil surveys were administered in person to employees. Upon reaching agreement on participation, the research team provided information sessions at each organizational location to describe the project, encourage participation, and address concerns from potential participants. Participation was voluntary, anonymous, and not rewarded. Members of the research team provided participants with informed consent materials that explained the anonymous nature of the data collection and their rights as research participants, and distributed the questionnaire. The majority of participants completed the survey that same day. In some instances, employees were allowed up to two weeks to complete the survey at home and return it in a sealed envelope to the research team that collected back the surveys on location. The data were collected at the end of 2018.

### Measures

Below is a description of measures used to provide data for the current analyses.

#### Emotional Contagion

Emotional contagion from the perspective of basic and discrete emotions absorbed by the respondent (i.e., EC absorbed) at the workplace was measured by the Emotional Contagion at Work Scale (ECWS; Petitta & Naughton, 2015). Previous findings support the empirical distinctiveness of contagion of the two discrete basic emotions assessed in this research, namely, joy and anger (Petitta & Naughton, 2015). The ECWS assesses emotional contagion at work by instructing respondents to focus on work-related situations wherein they happened to be involved in the emotional experiences described in the items. For example, a sample item from the 4-item joy-absorbed subscale is, “Interacting with happy people makes me feel better when I am a little down”, and a sample item from the 5-item anger-absorbed subscale is, “When someone is angry and raises their voice, I become irritated.” Participants were asked to indicate how frequently the emotional situation is experienced using a 5-point Likert scale ranging from 1 (*Never*) to 5 (*Always*)*.* Higher scores of “joy absorbed” and “anger absorbed” reflect greater levels of joy and anger being absorbed from others at work.

#### Sleep Disturbances

Sleep disturbances were measured using six items of the Karolinska Sleep Questionnaire (Kecklund & Åkerstedt, 1992). Participants were asked to indicate how frequently they experienced sleep disorders using a 5-point Likert scale ranging from 1 (*Never*) to 5 (*Very Often*)*.* A sample items is: “difficulties falling asleep” Higher scores of sleep disturbances reflect greater levels of problems associated to sleep.

#### Health Problems

Psychosomatic health problems of respondents was measured using Hanisch’s (1992) Health Complaints Index, a formative scale that tallies the total number of 13 health complaints (e.g., severe headaches, high blood pressure) experienced by respondents. Employees responded yes or no to these 13 health complaints. Higher numbers reflect more health problems ranging from 0 to 13.

#### Production Pressure

Organizational production pressure (Probst & Graso, 2013) was measured using five Likert-scale items. Participants indicated their agreement to the following items using response options ranging from 1 (*strongly disagree*) to 7 (*strongly agree*): “The main focus of this organization is on production. Everything else is second”.

#### Accidents at Work

The measure of experienced accidents was developed by Probst and Graso (2913) based on the U.S. Bureau of Labor Statistics’ Occupational Injury and Illness Classification System (OIICS; BLS, 2007). The OIICS is a formative scale and provides a classification system used to code precipitating events or exposures related to workplace illnesses and injuries. Probst and Graso (2013) developed a list of seventeen such exposures/events that were presented to employees who were asked to indicate (yes = 1/no = 0) if they had experienced each of the following events during the previous year, and if that exposure had resulted in either personal injury or property damage. The events included, for example: slip; trip. Thus, each employee’s *experienced events* score could range from 0 to 17.

#### Workplace Injuries

We used a 15-item self-report formative measure of workplace injuries (Probst et al., 2013) experienced during past year (e.g., back injury, cut/puncture). Workplace injuries were assessed by totaling the number of injuries workers indicated they had experienced as a result of their job (using a yes/no response scale), and could range from 0 to 15.

### Analytical Strategy

In order to maximize the balance between the number of subjects and the manifest indicators used for the implied SEM models, item parcels were created for construct measures with more than three items (i.e., joy-absorbed, anger-absorbed, production pressure, sleep disturbances). All latent variables were defined by three manifest indicators in order to get each latent variable just identified (Bollen, 1989). In the case of contagion dimensions, joy-absorbed latent variable was defined by one two-item parcel and two single-item indicators, while anger-absorbed was defined by two two-item parcels and one single-item indicator. Subsequent analyses were conducted with *Mplus* 8.0 (Muthén & Muthén, 1998–2017) using the parcels as manifest indicators of the latent variables. Moreover, in order to account for the skewed distribution of accidents at work and workplace injuries, the model was tested on the covariance matrix, using the Robust Maximum Likelihood estimation method (Yuan & Bentler, 2000).

Finally, in order to examine model fit, we used the following goodness-of-fit indices, as recommended by the literature (Byrne, 2006; Meade et al., 2008): Root Mean Square Error of Approximation (RMSEA), Comparative Fit Index (CFI), Tucker-Lewis index (TLI), and Standardized Root Mean Square Residual (SRMR). RMSEA is considered an absolute fit index that estimates lack of model fit and compensates for model complexity, with values of .05 or lower as indicating a well-fitting model, .05–.08 indicating a moderate fit, and .10 or greater indicating poor fit (Browne & Cudeck, 1993). The CFI and TLI are considered incremental fit indexes that compare the model of interest with a null or independence model (Bentler, 1990), with values of .90–.95 indicating acceptable fit and values above .95 indicating good fit (Hu & Bentler,1999). Finally, the SRMR estimates the discrepancy between the sample covariance matrix and the model covariance matrix, with values of .08 or lower as indicating a well-fitting model and values above .08 indicating poor fit (Hu & Bentler, 1999).

## Results

### Test of Measurement Model

In order to test our measurement model, we performed an initial confirmatory factor analysis with MPlus (Muthén & Muthén, 1998–2017). Since some parcels showed some slight departure from the univariate normal distribution, we tested our model with robust maximum likelihood estimation (Yuan & Bentler, 2000). The tested CFA model posited four continuous latent variables (i.e., joy-absorbed, anger-absorbed, production pressure, sleep disturbances) defined by their respective parcels as manifest indicators. Formative measures were not included (i.e., health problems, experienced accidents, and injuries). The model showed an excellent fit to the data: YBχ^2^_(*df* = 48, *N* = 1000)_ = 89.132, *p* < .001, RMSEA = .029 (.020; .039), CFI = .99, TLI = .98, SRMR = .025. Correlations among latent factors ranged from −.04 to .40, thus further supporting the distinctiveness of the study latent variables.

### Descriptive Statistics and Correlations

Table 1 presents the descriptive statistics, scale reliabilities, and intercorrelations among the study variables. Emotional contagion of anger (anger-contagion) was significantly and positively correlated with sleep disturbances and health problems (*r* = .20 and .22, respectively, *p* < .001), and with subsequent workplace accidents and injuries (*r* = .29 and .33, respectively, *p* < .001). Interestingly, emotional contagion of joy (joy-contagion) was weakly but significantly positively correlated with health problems (*r* = .09, *p* < .01). Yet, the correlation is extremely low in terms of magnitude and its statistical significance is mainly due to the large sample size (1000 subjects). Moreover, the percentage of variance that the two variables (contagion of joy, health problems) have in common is only less than 1% (i.e., .09^2^ = .0081). Hence, this counterintuitive finding could be considered as negligible.

**Table 1:** Descriptive statistics

Variable	M	SD	1	2	3	4	5	6	7
1. Emotional Contagion Joy	3.38	.91	*.77*						
2. Emotional Contagion Anger	2.60	.91	.31^**^	*.82*					
3. Production Pressure	3.08	1.34	−.02	.13^**^	*.77*				
4. Sleep Disturbances	2.88	.73	−.02	.20^**^	.19^**^	*.84*			
5. Health Problems	2.74	2.34	.09^**^	.22^**^	.19^**^	.51^**^	*–*		
6. Accidents	2.32	2.63	−.03	.17^**^	.29^**^	.26^**^	.30^**^	*–*	
7. Injuries	1.99	2.20	.01	.17^**^	.33^**^	.27^**^	.36^**^	.58^**^	*–*

Note: ^**^p < .01; Cronbach’s alpha reliability coefficients are on the diagonal

Sleep disturbances and health complaints were both significantly and positively correlated with workplace accidents (*r* = .26 and .27, respectively, *p* < .001), and injuries (*r* = .30 and .36 respectively, *p* < .001). Moreover, higher levels of production pressure were associated with increased sleep disturbances and health complaints (*r* = .19 and .19, respectively, *p* < .001). Finally, contagion of joy was significantly and positively correlated with contagion of anger (*r* = .31, *p* < .01). While contagion of joy measures the perceived susceptibility to absorb a positive emotion and contagion of anger measures the perceived susceptibility to absorb a negative emotion, the two variables both measure people’s perception of their susceptibility to catch other people’s emotions. As such, the positive correlation reflects the common latent factor of “contagion as perceived susceptibility”.

### Test of Structural Model

Consistent with Klein and Moosbrugger’s (2000) recommendations, we used a two-step approach in order to test the posited moderated mediation model. First, we tested a model without including the latent interactions posited in Fig. 1 (Model 0), and using the robust maximum likelihood estimators recommended for skewed observed variables (Yuan & Bentler, 2000). This model showed good fit to the data: YBχ^2^_(*df* = 73, *N* = 1000)_ = 209.239, *p* < .001, RMSEA = .043 (90% C.I. .036–.050), CFI = .97, TLI = .96, SRMR = .04. Next, we tested the posited model including the latent interaction terms (Model 1) estimated with numerical integration. Since the overall fit of Model 1 cannot be evaluated with commonly used fit indices (see Muthén & Asparouhov, 2015), it was statistically compared with Model 0 by means of the log-likelihood ratio test *D* (Maslowsky, Jager, & Hemken, 2015), where *D* = −2[(log-likelihood for Model 0) – (log-likelihood for Model 1)] and the number of degrees of freedom is calculated as the difference between Model 0 and Model 1 estimated parameters. Results showed a significant D (*D*_(df = 4)_ = 36.90, *p* < .001), thus demonstrating that our hypothesized model positing the latent interactions can be retained as the final structural model.

**Fig. 2:**
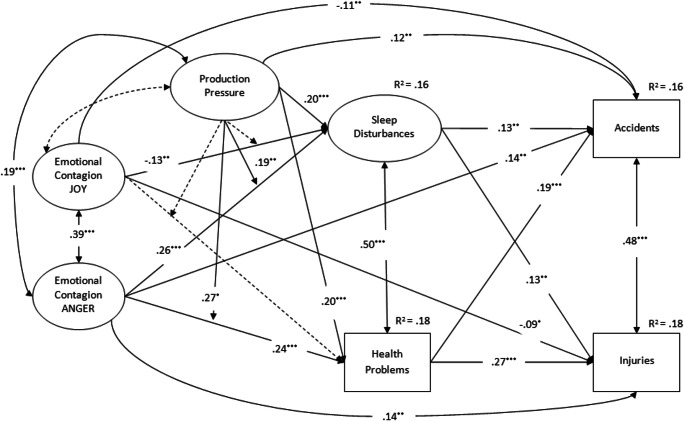
Standardized structural coefficients for the structural model. *Note*. ^***^p < .001, ^**^p < .01, ^*^p < .05; dotted lines are statistically non-significant estimates

Figure 2 shows the standardized coefficients for the final structural model examining the moderating role of production pressure in the relationships between emotional contagion (of joy and anger) and sleep disturbances as well as health problems, which in turn predict workplace accidents and injuries. Consistent with our proposed conceptual model positing both production pressure and contagion of anger as job demands and contagion of joy as a job resource, production pressure may be considered a same level predictor along with emotional contagion of anger and joy. As such, in our model we have specified production pressure as a covariate of both emotional contagion of anger and emotional contagion of joy.

As can be seen, sleep disturbances was associated with higher number of accidents (.13, *p* < .01) and injuries (.13, *p* < .01). Similarly, health problems was associated with higher number of accidents (.19, *p* < .001) and injuries (.27, *p* < .001). Both of these findings lend support to Hypotheses 1 and 2. Moreover, in general support of Hypotheses 3 and 4, joy-contagion negatively associated with sleep disturbances (−.13, *p* < .01) whereas it did not exert a significant effect on health problems. Anger-contagion was positively associated with both sleep disturbances (.26, *p* < .001) and health problems (.24, *p* < .001). Furthermore, joy-contagion negatively associated with both workplace accidents (−.11, *p* < .01) as well as injuries (−.09, *p* < .05), while anger-contagion positively associated with both workplace accidents (.14, *p* < .01) as well as injuries (.14, *p* < .01). Additionally, because both joy-contagion and anger-contagion remained significant predictors of safety outcomes even after accounting for employee sleep disturbances and health problems, this lends support to the partial mediating effect of overall health-related problems (i.e., sleep disturbances, health problems).

**Fig. 3:**
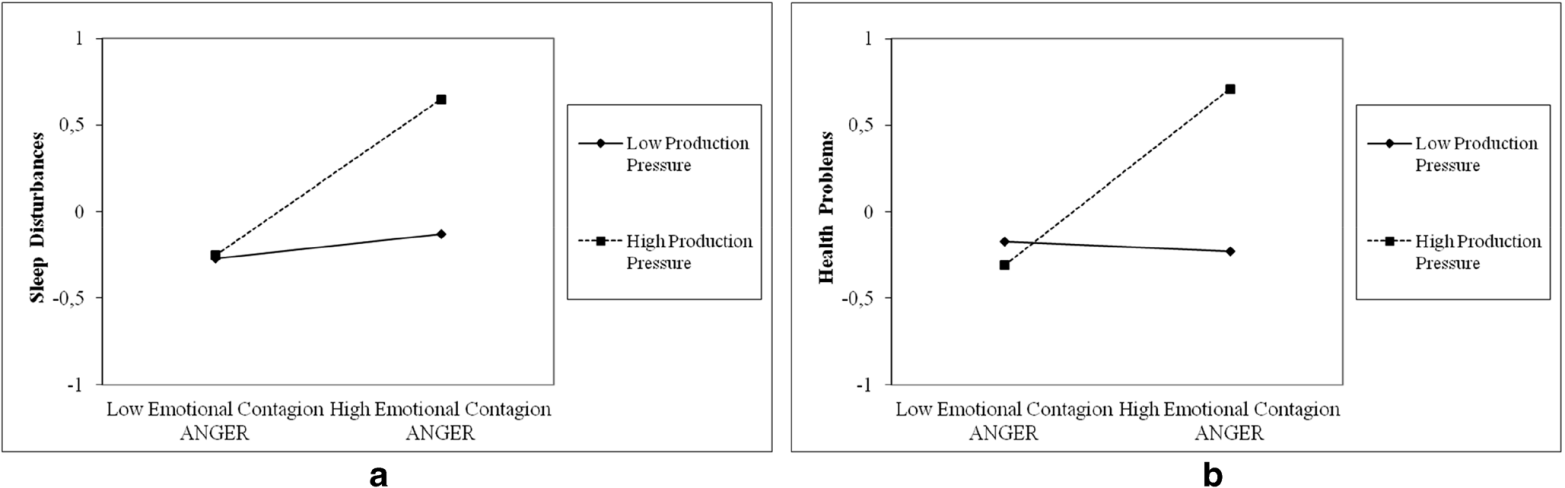
Moderating effect of production pressure on the relationship between emotional contagion of anger and sleep disturbances (**a**), and emotional contagion of anger and health problems (**b**)

Production pressure demonstrated significant main positive effects on both sleep disturbances (.20, *p* < .001) and health problems (.20, *p* < .001), such that higher levels of production pressure associated with increased sleep disturbances and poorer health, thus providing support for Hypothesis 5. Moreover, production pressure significantly associated with higher number of accidents (.12, *p* < .01). Additionally, as predicted by Hypotheses 6a and 6b, the multiplicative effect of production pressure and anger-contagion exerted a significant effect on both sleep disturbances (.19, *p* < .001) and health problems (.27, *p* < .05), such that production pressure exacerbated the impact of anger-contagion on both employee sleep disturbances and health problems. Conversely, no significant interactive effect between production pressure and joy-contagion on both sleep disturbances and health problems were detected, thus failing to support Hypotheses 6c and 6d. Figure 3a and b illustrate the form of the interaction between contagion of anger and production pressure. As can be seen, the relationship between contagion of anger and sleep disturbances as well as contagion of anger and health problems are stronger under conditions of high (+1 SD) production pressure, rather than low (−1 SD) production pressure.

Finally, we examined the specific indirect effects assessing their 95% bias-corrected confidence intervals (BCCIs) estimated across 1000 bootstrap samples (MacKinnon et al., 2004). Given that our final model posited latent interaction effects, numerical integration was used also to estimate the specific indirect effects (Cheung & Lau, 2017). Results showed that joy-contagion exerted a negative effect on accidents via sleep disturbances (−.05, CI: −.09 − −.01), but not via health problems. Similarly, joy-contagion exerted a negative effect on injuries via sleep disturbances (−.04, CI: −.08 − −.004), but not via health problems. Anger-contagion exerted a positive effect on accidents via sleep disturbances (.09, CI: .03–.16) and health problems (.12, CI: .05–.19). Similarly, anger-contagion exerted a positive effect on injuries via sleep disturbances (.07, CI: .02–.13) and health problems (.14, CI: .07–.23). As such, Hypotheses 3 and 4 on indirect effects were generally confirmed.

Overall, the model explained the 16% of variance in accidents and the 18% of the variance in injuries. Furthermore, the combined emotional contagion and production pressure factors explained 16% of the variance in sleep disturbances and 18% of the variance in health problems.

## Discussion

Workers around the globe annually experience approximately 260 million occupational injuries and 350,000 fatalities due to work-related injuries (Hämäläinen et al., 2006). According to the Italian National Workers Compensation Authority (INAIL, 2016), nearly 640,000 work-related injuries occurred in Italy. Meta-analytic findings suggest that approximately 13% of work injuries could be attributed to sleep problems (Kuehli et al., 2014). Furthermore, according to recent evidence (Hafner et al., 2016), the proportion of people getting less than the recommended hours of sleep is rising worldwide.

Despite the growing body of evidence linking poor sleep and health with adverse safety outcomes, no research to date has examined whether emotional contagion at work accounts for variance in the occurrence of sleep disturbances, ill health, and work-related safety outcomes. The current study fills this gap by investigating the concomitant effects of workplace contagion of both positive and negative emotions (i.e., joy, anger) on the perceived levels of sleep and health problems as well as the occurrence of workplace accidents and injuries. Furthermore, the research aimed to test the extent to which the relationships between emotional contagion and poor sleep and health may be strengthened or weakened as a function of perceived production pressure.

Our findings suggest that poor work-related health is a stronger explanatory variable of accidents and injuries than sleep disturbances; yet, it does not mediate the effects of contagion of joy on poor safety outcomes. Rather, sleep disturbances act as the primary mediator between contagion of both positive and negative emotions and poor safety outcomes. Such results highlight the importance of considering not only sleep disturbances but also other psychosomatic work-related health problems when investigating workplace accidents and injuries. Specifically, when taking into account both types of physiological problems, our results suggest that health problems may be more impactful on accidents and injuries, whereas sleep disturbances may be the more operative factor explaining the impact of emotional contagion at work on safety outcomes.

Furthermore, our results showed that greater contagion of anger at work was associated with more sleep disturbances and ill health, whereas contagion of joy did not explain psychosomatic health problems. Hence, when employees absorb more anger from workplace interactions they not only experience more sleep and health problems, but also more accidents and injuries at work. Conversely, employee absorption of joy (a job resource) may help in preventing the occurrence of sleep disturbances (a negative variable), but not health problems or safety outcomes.

As such, the differential role of emotional contagion of anger and joy for sleep and health seems to suggest that insufficient sleep may be an antecedent of negative health outcomes (e.g., Hafner et al., 2016) rather than a symptom of underlying health problems (e.g., National Sleep Foundation, 2018). Additionally, the dominant role played by contagion of anger at work in comparison to the contagion of joy is consistent with meta-analytic findings (Litwiller et al., 2017) suggesting that higher trait negative affect relates to worse sleep quality.

This may be particularly relevant during the current pandemic spread of Coronavirus disease (COVID-19). In addition to reducing transmission of the virus itself, it is also important to reduce transmission of negative emotions which may compromise worker safety and health. During the pandemic, many employees are working under more stressful situations; as tensions rise and lead to anger, this could spread within the team leading to increased likelihood of making a mistake (Brennan & Oeppen, 2020).

More interestingly, the indirect effects of emotional contagion of anger on safety outcomes via sleep and health problems were intensified as levels of pressure increased. That is, employees who perceived higher levels of production pressure were even more sensitive to the effects of anger absorbed through social exchanges at work, thus reporting more sleep disturbances and health problems, and more accidents and injuries. As such, organizations with high levels of production pressure may expect not only higher occurrence of accidents and injuries when anger spreads among employees at the workplace, but also worse employee physiological conditions (i.e., sleep disturbances and health problems). Overall, our findings suggest the relevance of examining both individual and contextual factors when studying the link between employee sleep and health problems and poor safety outcomes.

### Theoretical Implications for Sleep, Health, and Emotional Contagion

Together, our findings have implications for the extant literature in the areas of sleep, occupational health, safety, and emotional contagion. First, our study contributes to bridge the gap between the fields of organizational psychology and sleep research, which still lack conclusive knowledge about the relationship between sleep and work (Litwiller et al., 2017). In doing so, we add to sleep and organizational health research by including both individual (i.e., emotional contagion of joy/anger) and contextual (i.e., organizational production pressure) factors that influence employee sleep disturbances and health problems, and related workplace accidents and injuries. Specifically, we extended meta-analytic findings by Nahrgang et al. (2011) and found empirical support for the conceptual frameworks of Krauss et al. (2003) and Mullins et al. (2014) which posited effects of job demands and resources on employee health and safety outcomes. Namely, we demonstrated that the job-related demands of emotional contagion of anger and production pressure are associated with increased sleep disturbances and health problems, and more work-related safety issues.

Thus, it appears that anger absorbed from others at work and the organizational pressure to produce may impede healthy physiological functioning during sleep, i.e., the switch from action-related sympathetic to calm-related parasympathetic nervous system which is necessary to restoring energies that prevent health problems. Interestingly, consistent with the growing relevance of positive psychology (Fredrickson, 2001) in the workplace, in our study the *contagion* of joy at work (i.e., a *social* source of emotion) appears to prevent the likelihood of sleep disturbances, similar to other studies examining the effect of *intra-individual* happiness and sleep (Ong et al., 2013). That is, not only do “first-hand” positive emotions help employee to avoid sleep disorders, but also “second-hand” positive emotions that originate socially and are absorbed from others at work may exert similar effects.

Second, the results of this research also inform the safety literature by demonstrating the combined impact of organizational production pressure and emotional contagion of anger on sleep and health problems. Prior research (e.g., Dement & Vaughan, 1999; Hafner et al., 2016) has found support for the role of organizational time pressure in predicting employee sleep and health problems. Our findings build upon this previous work by also suggesting production pressure can exacerbate the effects of contagion of anger absorbed by employees at work on their sleep and health problems. That is, contagion of anger among employees can lead to greater accidents and injuries as a result of insufficient sleep and health problems, and this effect can be further strengthened by organizational production demands.

We also extend previous theorizing about emotional contagion by incorporating sleep disturbances, health problems and safety. While literature has established the role of emotional contagion in shaping employee well-being (e.g., job burnout; Le Blanc et al., 2001), the current study is the first to include emotion-related factors (i.e., emotional contagion), in addition to production pressure, as explanatory mechanisms of restorative sleep and healthy behaviors.

Finally, our findings contribute to expanding the Job Demands-Resources model of stress and safety (Demerouti et al., 2004; Kühnel et al., 2012; Nahrgang et al., 2011) by examining emotion-related job demands (i.e., emotional contagion of anger) and job resources (i.e., emotional contagion of joy) that employees cannot easily “turn off” once they go home (Demerouti et al., 2004). Moreover, we examined how an individual-related job demand (i.e., contagion of anger) interacts with an organizational demand (i.e., production pressure) in altering employee self-reported psycho-physiological functioning (i.e., sleep and health problems) and their ability to work safely. These results comport with Bakker and Demerouti’s (2017) assertion that different job demands interact and exert a cumulative effect on employee wellbeing.

### Practical Implications for Employees and Organizations

From a practical perspective, the results of this study have important implications. Not only are insufficient sleep rates on the rise in all industrialized countries, but also the economic costs of the related consequences in terms of workplace accidents and injuries are a worldwide concern (Hafner et al., 2016). Previous research has shown that job demands interfere with proper employee recovery at home, leading to burnout and subsequent unsafe behaviors (Demerouti et al., 2004; Nahrgang et al., 2011). In a similar fashion, our results warn organizations of loss spirals that may occur due to disturbed sleep and health-related problems attributable to emotional contagion at work. Specifically, rather than of seeking to remedy poor sleep and health, organizations might more effectively focus on the prevention of these issues by reducing anger in the workplace and increasing joy. By doing so, they may also observe positive downstream effects on workplace accidents and injuries.

For example, supervisors play a key role in affecting employees’ emotions at work and related health and performance. According to Jia and Cheng (2020), supervisor immediacy in expressing positive emotions affects employees’ sense of psychological safety, positive emotions toward others and enjoyment of work through emotional contagion mechanisms. This contributes to generate satisfaction for supervisor-subordinate communication and to create meaning-laden social processes that can improve employee job performance as well as health and wellbeing. As such, supervisors’ efforts to create comfortable and reliable relationship dynamics through the spread of positive emotional processes may influence overall employee work experiences and job engagement.

Intervention programs might also provide management and employees with tools to help them augment the experience of absorption of joy, and conversely inhibit the experience of absorbing other’s anger. For example, the experience of joy can be facilitated with gratitude exercises where one thinks of things that happened during the workday to be grateful for and records them in writing (Boyatzis et al., 2015). Moreover, emotion management techniques such as mindfulness also have been shown to be effective in alleviating the absorption of negative emotions by reducing rumination (Sanders & Lam, 2010) and, therefore, eliciting a salubrious state of restorative relaxation that facilitates sleep and healthy life conditions (Christensen et al., 2004).

Finally, effective emotion management builds upon employees self-awareness of emotional processes as a first step to recognize how one’s social interactions with other people at work contribute to feelings of joy and anger, as well as the mechanisms through which these emotions may cause one to experience high/low problems in taking restorative sleep and performing safety in the context of organizational time and production pressure. Specifically, interventions aimed at preventing sleep and health problems and workplace accidents may target employees and focus on gaining knowledge about the nature of emotions Andrieş (2011) and developing skills to manage their emotional resources so as to adapt to job requirements and work while preserving health and increasing organizational effectiveness and safety. In some instances, this may require a reframe of organizational thinking and, therefore, a change in the company culture, and the culture of safety in particular (Petitta et al., 2017). Additionally, employers may provide facilities and amenities that help employees with sleep hygiene, such as putting in place arrangements to support their staff’s daily routines, and/or discourage the extended use of electronic devices or signal limits on staff’s expected availability after working hours.

### Strengths, Limitations, and Future Directions

The current study is the first to demonstrate that production pressure interacts with contagion of emotions at work in explaining employee sleep and health problems and subsequent accidents and injuries. While our findings are promising and shed light on the interplay between individual (i.e., emotional contagion) and contextual (i.e., production pressure) factors affecting employees’ physiological problems and subsequent poor safety outcomes, they also warrant further investigation. First, our hypotheses were tested using self-report data, thus raising the likelihood of mono-method bias (Podsakoff et al., 2003). Notably, however, previous research indicates that self-report measures of accidents and unsafe behaviors are related to independent observations of these variables (Lusk et al., 1995), thus reducing the likelihood that mono-method bias may be an issue in the present study. Nevertheless, future studies may seek secondary-source assessment of accidents and injuries (e.g., from supervisors) in an attempt to overcome the potential issues caused by common method variance, and provide added support for the causal links posited in our model. Additionally, sleep quality and quantity could be investigated by using personal physical fitness trackers (e.g., FitBit, Apple watch) to collect objective physiological data on the overall amount of time slept, the length of time it takes to fall asleep, and the number of times the person was restless or awoke during the night.

Second, the current study relies on cross-sectional data. Future research should replicate our model using longitudinal research where the predictors are measured at Time 1, mediators and moderators are measured at Time 2, and outcomes are measured at Time 3. Third, in addition to conducting longitudinal studies with multiple data sources, future work can delineate the influence of contagion of additional discrete emotions. In this study we focused on joy and anger as positive and negative emotions. Given that literature (Gilboa et al., 2008) suggests that negative emotions (i.e., frustration, anxiety) increase the number of accidents experienced by employees, future studies may consider the impact of contagion of other discrete emotions (e.g., sadness, fear) on employee health and subsequent carrying out of work in a safe manner.

Fourth, while we focused on sleep and health problems as the mediators in linking emotional contagion at work and poor safety outcomes, future research may explore other mechanisms related to employee wellbeing (e.g., affective wellbeing, mental health) underlying the relationship between emotional contagion and safety outcomes in general and the contagion of anger/joy and safety in particular (because of the partial mediation relationship). Finally, an additional venue for advancing the extant literature is to incorporate additional contextual effects of organizational processes into the proposed conceptual model, such as safety climate (Bronkhorst, 2015) and safety culture (Petitta et al., 2017), and examine how organizational factors may contribute to shaping employees emotional life and its impact on their wellbeing and related likelihood of incurring into accidents and injuries. Toward that end, future studies may consider possible organizational differences, take a multilevel modeling approach, and target employees within a wide variety of organizations.

## Data Availability

The datasets generated during and/or analysed during the current study are available from the corresponding author on reasonable request.

## Footnotes

1 Any use of the word “predictor” in our study is strictly meant in the statistical sense, rather than a causal one.

## References

[CR1] Andrieş AM (2011). Positive and negative emotions within the organizational context. Global Journal of Human Social Science.

[CR2] Bakker AB, Demerouti E (2007). The job demands-resources model: State of the art. Journal of Managerial Psychology.

[CR3] Bakker AB, Demerouti E (2017). Job demands–resources theory: Taking stock and looking forward. Journal of Occupational Health Psychology.

[CR4] Barling, J. E., & Frone, M. R. (2004). *The psychology of workplace safety*. American Psychological Association.

[CR5] Barnes CM (2012). Working in our sleep: Sleep and self-regulation in organizations. Organizational Psychology Review.

[CR6] Barsade SG (2002). The ripple effect: Emotional contagion and its influence on group behavior. Administrative Science Quarterly.

[CR7] Bentler PM (1990). Comparative fit indexes in structural models. Psychological Bulletin.

[CR8] Bollen, K. A. (1989). *Structural equations with latent variables*. John Wiley & Sons.

[CR9] Boyatzis R, Rochford K, Taylor S (2015). The role of the positive emotional attractor in vision and shared vision: Toward effective leadership, relationships, and engagement. Frontiers in Psychology.

[CR10] Brennan PA, Oeppen RS (2020). Editorial: Safe healthcare teams during the coronavirus outbreak. The British Journal of Oral & Maxillofacial Surgery.

[CR11] Bronkhorst B (2015). Behaving safely under pressure: The effects of job demands, resources, and safety climate on employee physical and psychosocial safety behavior. Journal of Safety Research.

[CR12] Browne, M. W., & Cudeck, R. (1993). Alternative ways of assessing model fit. In K. A. Bollen & J. S. Long (Eds.), *Testing structural equation models* (pp. 136–162). Sage.

[CR13] Bureau of Labor Statistics. (2007*). Occupational injury and illness classification manual 2007*. Retrievable at: https://www.bls.gov/iif/oiics_manual_2007.pdf

[CR14] Burgard SA, Ailshire JA (2009). Putting work to bed: Stressful experiences on the job and sleep quality. Journal of Health and Social Behavior.

[CR15] Byrne, B. M. (2006). *Structural Equation Modeling with EQS: Basic Concepts, Applications,and Programming* (2nd ed.). Lawrence Erlbaum Associates.

[CR16] Cheung GW, Lau RS (2017). Accuracy of parameter estimates and confidence intervals in moderated mediation models: A comparison of regression and latent moderated structural equations. Organizational Research Methods.

[CR17] Christensen, A. J., Martin, R., & Smyth, J. M. (2004). *Encyclopedia of Health Psychology*. Springer.

[CR18] Dement, W. C., & Vaughan, C. C. (1999). *The promise of sleep: A Pioneer in sleep medicine explores the vital connection between health, happiness, and a good Night's sleep*. Delacorte Press.

[CR19] Demerouti E, Bakker AB, Bulters AJ (2004). The loss spiral of work pressure, work-home interference and exhaustion: Reciprocal relations in a three-wave study. Journal of Vocational Behavior.

[CR20] Doherty RW (1997). The emotional contagion scale: A measure of individual differences. Journal of Nonverbal Behavior.

[CR21] Ekman, P. (1999). Basic emotions. In T. Dalgleish & T. Power (Eds.), *The handbook of cognition and emotion* (pp. 45–60). John Wiley & Sons, Ltd..

[CR22] European Agency for Safety and Health at Work (2019). *The economics of occupational safety and health – The value of OSH to society*. Retrievable at: https://visualisation.osha.europa.eu/osh-costs#!/.

[CR23] Fredrickson BL (2001). The role of positive emotions in positive psychology: The broaden-and-build theory of positive emotions. American Psychologist.

[CR24] Gallie D (2005). Work pressure in Europe 1996-2001: Trends and determinants. British Journal of Industrial Relations.

[CR25] Gilboa S, Shirom A, Fried Y, Cooper CL (2008). A meta-analysis of work demand stressors and job performance: Examining main and moderating effects. Personnel Psychology.

[CR26] Hafner, M., Stepanek, M., Taylor, J., Troxel, W. T., & van Stolk, C. (2016). *Why sleep matters –the economic costs of insufficient sleep. A cross-country comparative analysis*. RAND Corporation. 10.7249/rhq-06-04-11 PMC5627640 28983434

[CR27] Hämäläinen P, Takala J, Saarela K (2006). Global estimates of occupational accidents. Safety Science.

[CR28] Han S, Saba F, Lee S, Mohamed Y, Peña-Mora F (2014). Toward an understanding of the impact of production pressure on safety performance in construction operations. Accident Analysis and Prevention.

[CR29] Hanisch KA (1992) The development of a health condition scale and its relations to health satisfaction and retirement valence. In: *2nd APA.NIOSH Conference on Stress and the Workplace*, Washington, DC, November 1992.

[CR30] Hatfield E, Cacioppo JT, Rapson RL (1993). Emotional contagion. Current Directions in Psychological Science.

[CR31] Hatfield E, Forbes M, Rapson RL (2012). Marketing love and sex. Society.

[CR32] Hatfield, E., & Rapson, R. L. (1998). Emotional contagion and the communication of emotions. In M. T. Palmer & G. A. Barnett (Eds.), *Progress in communication sciences* (Vol. 14, pp. 73–89). Ablex.

[CR33] Health & Safety Executive. (2018). What are the Management Standards? Retrievable at: http://www.hse.gov.uk/stress/standards/

[CR34] Hess, U., & Fischer, A. (2014). *Emotional mimicry in social context*. Cambridge University Press.

[CR35] Hu L, Bentler PM (1999). Cutoff criteria for fit indexes in covariance structure analysis: Conventional criteria versus new alternatives. Structural Equation Modeling.

[CR36] Iacoboni M (2009). Imitation, empathy, and mirror neurons. Annual Review of Psychology.

[CR37] Italian National Workers Compensation Authority. (2016). Biannual analysis of the number of accidents. Available at: http://dati.inail.it/opendata_files/downloads/daticoncadenzasemestraleinfortuni/Tabelle_nazionali_cadenza_semestrale.pdf

[CR38] Jia, M., & Cheng, J. (2020). Emotional experiences in the Workplace: Biological Sex, Supervisor Nonverbal Behaviors, and Subordinate Susceptibility to Emotional Contagion. *Psychological Reports*. 10.1177/0033294120940552. 10.1177/0033294120940552 32635815

[CR39] Kaminski M (2001). Unintended consequences: Organizational practices and their impact on workplace safety and productivity. Journal of Occupational Health Psychology.

[CR40] Kecklund G, Åkerstedt T (1992). The psychometric properties of the Karolinska sleep questionnaire. Journal of Sleep Research.

[CR41] Keren N, Mills TR, Freeman SA, Shelley MI (2009). Can level of safety climate predict level of orientation toward safety in a decision making task?. Safety Science.

[CR42] Klein A, Moosbrugger H (2000). Maximum likelihood estimation of latent interaction effects with the LMS method. Psychometrika.

[CR43] Krauss, D. A., Chen, P. Y., De Armond, S., & Moorcroft, B. (2003). Sleepiness in the workplace: Causes, consequences, and countermeasures. In C. L. Cooper & I. T. Robertson (Eds.), *International review of industrial and organizational psychology* (Vol. 8, pp. 81–129). Wiley.

[CR44] Kuehli K, Mehta AJ, Miedinger D, Hug K, Schindler C, Holsboer-Trachsler E, Leuppi JD, Künzli N (2014). Sleep problems and work injuries: A systematic review and meta-analysis. Sleep Medicine Reviews.

[CR45] Kühnel J, Sonnentag S, Bledow R (2012). Resources and time pressure as day-level antecedents of work engagement. Journal of Occupational and Organizational Psychology.

[CR46] Le Blanc, P. M., Bakker, A. B., Peeters, M. C. W., van Heesch, Nicolette C. A, & Schaufeli, W. B. (2001). Emotional job demands and burnout among oncology care providers. Anxiety, Stress, and Coping*,* 14(3), 243–263. 10.1080/10615800108248356.

[CR47] LeDoux, J. (2002). *Synaptic self: How our brains become who we are*. Viking.

[CR48] Leger D, Poursain B, Neubauer D, Uchiyama M (2008). An international survey of sleeping problems in the general population. Current Medical Research and Opinion.

[CR49] Litwiller B, Snyder LA, Taylor WD, Steele LM (2017). The relationship between sleep and work: A meta-analysis. Journal of Applied Psychology.

[CR50] Liu Y, Wheaton AG, Chapman DP, Cunningham TJ, Lu H, Croft JB (2016). Prevalence of healthy sleep duration among adults — United States 2014. MMWR. Morbidity and Mortality Weekly Report.

[CR51] Lusk S, Ronis D, Baer L (1995). A comparison of multiple indicators: Observations, supervisor report, and self-report measures of worker’s hearing protection use. Evaluation & the Health Professions.

[CR52] MacKinnon DP, Lockwood CM, Williams J (2004). Confidence limits for the indirect effect: Distribution of the product and resampling methods. Multivariate Behavioral Research.

[CR53] Magnusson Hanson LL, Chungkham HS, Åkerstedt T, Westerlund H (2014). The role of sleep disturbances in the longitudinal relationship between psychosocial working conditions, measured by work demands and support, and depression. Sleep.

[CR54] Martin M (1983). Cognitive failure: Everyday and laboratory performance. Bulletin of the Psychonomic Society.

[CR55] Maslowsky J, Jager J, Hemken D (2015). Estimating and interpreting latent variable interactions: A tutorial for applying the latent moderated structural equations method. International Journal of Behavioral Development.

[CR56] Meade AW, Johnson EC, Braddy PW (2008). Power and sensitivity of alternative fit indices in tests of measurement. Journal of Applied Psychology.

[CR57] Meijman, T. F., & Mulder, G. (1998). Psychological aspects of workload. In P. D. Drenth, H. Thierry, C. J. de Wolff, P. D. Drenth, H. Thierry, C. J. de Wolff (Eds.), *Handbook of work and organizational: Work psychology* (pp. 5–33). Psychology Press/Erlbaum (UK) Taylor & Francis.

[CR58] Mullins HM, Cortina JM, Drake CL, Dalal RS (2014). Sleepiness at work: A review and framework of how the physiology of sleepiness impacts the workplace. Journal of Applied Psychology.

[CR59] Muthén, B., & Asparouhov, T. (2015, November 2). Latent variable interactions. Retrieved from https://www.statmodel.com/download/LVinteractions.pdf

[CR60] Muthén, L. K., & Muthén, B. O. (1998-2017). *Mplus User's Guide,* (8th ed.) Muthén & Muthén.

[CR61] Nahrgang JD, Morgeson FP, Hofmann DA (2011). Safety at work: A meta-analytic investigation of the link between job demands, job resources, burnout, engagement, and safety outcomes. Journal of Applied Psychology.

[CR62] National Sleep Foundation. (2013). *International Bedroom Poll*. As of 28 November 2016: https://sleepfoundation.org/sleep-polls-data/other-polls/2013-international-bedroom-poll

[CR63] National Sleep Foundation. (2018). *Sleep, performance & the workplace*. Retrievable from: https://sleepfoundation.org/sites/default/files/sleepcarecenters/Sleep_Performance_the_Workplace.ppt.

[CR64] Nuckols TK, Bhattacharya J, Wolman D, Ulmer C, Escarce JJ (2009). Cost implications of reduced work hours and workloads for resident physicians. New England Journal of Medicine.

[CR65] Ong AD, Exner-Cortens D, Riffin C, Steptoe A, Zautra A, Almeida DM (2013). Linking stable and dynamic features of positive affect to sleep. Annals of Behavioral Medicine : A Publication of the Society of Behavioral Medicine.

[CR66] Pack AI, Pack AM, Rodgman E, Cucchiara A, Dinges DF, Schwab CW (1995). Characteristics of crashes attributed to the driver having fallen asleep. Accident Analysis & Prevention.

[CR67] Petitta L, Naughton S (2015). Mapping the association of emotional contagion to leaders, colleagues, and clients: Implications for leadership. Organization Management Journal.

[CR68] Petitta L, Probst TM, Barbaranelli C, Ghezzi V (2017). Disentangling the roles of safety climate and safety culture: Multi-level effects on the relationship between supervisor enforcement and safety compliance. Accident Analysis and Prevention.

[CR69] Petitta L, Probst TM, Ghezzi V, Barbaranelli C (2019). Cognitive failures in response to emotional contagion: Their effects on workplace accidents. Accident Analysis and Prevention.

[CR70] Podsakoff PM, MacKenzie SB, Lee JY, Podsakoff NP (2003). Common method biases in behavioral research: A critical review of the literature and recommended remedies. Journal of Applied Psychology.

[CR71] Probst TM, Graso M (2013). Pressure to produce=pressure to reduce accident reporting?. Accident Analysis and Prevention.

[CR72] Probst TM, Graso M, Estrada AX, Greer S (2013). Consideration of future safety consequences: A new predictor of employee safety. Accident Analysis & Prevention.

[CR73] Rizzolatti, G., & Sinigaglia, C. (2008). *Mirrors in the brain: How our minds share actions and emotions*. Oxford University Press Inc..

[CR74] Rundmo T, Hestad H, Ulleberg P (1998). Organisational factors, safety attitudes and workload among offshore oil personnel. Safety Science.

[CR75] Sanders WA, Lam DH (2010). Ruminative and mindful self-focused processing modes and their impact on problem solving in dysphoric individuals. Behaviour Research and Therapy.

[CR76] Shoss, M. K., & Probst, T. M. (2012). Multilevel outcomes of economic stress: An agenda for future research. In P. L. Perrewé, J. R. B. Halbesleben, & C. C. Rosen (Eds.), *The role of the economic crisis on occupational stress and well-being: Research in occupational stress and well-being* (Vol. 10, pp. 43–86). Emerald Group Publishing Limited.

[CR77] Silla I, Gamero N (2013). Shared time pressure at work and its health-related outcomes: Job satisfaction as a mediator. European Journal of Work and Organizational Psychology.

[CR78] Vgontzas AN, Liao D, Pejovic S, Calhoun S, Karataraki M, Basta M, Fernández-Mendoza J, Bixler EO (2010). Insomnia with short sleep duration and mortality: The Penn State cohort. Sleep.

[CR79] Yuan, K. H., & Bentler, P. M. (2000). Three likelihood-based methods for mean and covariance structure analysis with nonnormal missing data. In M. E. Sobel & M. P. Becker (Eds.), *Sociological methodology 2000* (pp. 165–200). ASA.

[CR80] Zohar D, Luria G (2005). A multilevel model of safety climate: Cross-level relationships between organization and group-level climates. Journal of Applied Psychology.

